# The evolution of antennal courtship in diplazontine parasitoid wasps (Hymenoptera, Ichneumonidae, Diplazontinae)

**DOI:** 10.1186/1471-2148-10-218

**Published:** 2010-07-20

**Authors:** Seraina Klopfstein, Donald LJ Quicke, Christian Kropf

**Affiliations:** 1 Natural History Museum (Invertebrates), Bernastrasse 15, CH-3005 Bern, Switzerland; 2 Division of Community Ecology, Institute of Ecology and Evolution, University of Bern, Baltzerstr. 7, CH-3012 Bern, Switzerland; 3 Division of Biology, Imperial College London, Silwood Park Campus, Ascot, Berkshire SL5 7PY, UK; 4 Department of Entomology, Natural History Museum, London SW7 5BD, UK

## Abstract

**Background:**

As predicted by theory, traits associated with reproduction often evolve at a comparatively high speed. This is especially the case for courtship behaviour which plays a central role in reproductive isolation. On the other hand, courtship behavioural traits often involve morphological and behavioural adaptations in both sexes; this suggests that their evolution might be under severe constraints, for instance irreversibility of character loss. Here, we use a recently proposed method to retrieve data on a peculiar courtship behavioural trait, i.e. antennal coiling, for 56 species of diplazontine parasitoid wasps. On the basis of a well-resolved phylogeny, we reconstruct the evolutionary history of antennal coiling and associated morphological modifications to study the mode of evolution of this complex character system.

**Results:**

Our study reveals a large variation in shape, location and ultra-structure of male-specific modifications on the antennae. As for antennal coiling, we find either single-coiling, double-coiling or the absence of coiling; each state is present in multiple genera. Using a model comparison approach, we show that the possession of antennal modifications is highly correlated with antennal coiling behaviour. Ancestral state reconstruction shows that both antennal modifications and antennal coiling are highly congruent with the molecular phylogeny, implying low levels of homoplasy and a comparatively low speed of evolution. Antennal coiling is lost on two independent occasions, and never reacquired. A zero rate of regaining antennal coiling is supported by maximum parsimony, maximum likelihood and Bayesian approaches.

**Conclusions:**

Our study provides the first comparative evidence for a tight correlation between male-specific antennal modifications and the use of the antennae during courtship. Antennal coiling in Diplazontinae evolved at a comparatively low rate, and was never reacquired in any of the studied taxa. This suggests that the loss of antennal coiling is irreversible on the timescale examined here, and therefore that evolutionary constraints have greatly influenced the evolution of antennal courtship in this group of parasitoid wasps. Further studies are needed to ascertain whether the loss of antennal coiling is irreversible on larger timescales, and whether evolutionary constraints have influenced courtship behavioural traits in a similar way in other groups.

## Background

Empirical and theoretical evidence suggests that traits linked to reproduction evolve more rapidly than other traits and might be involved in speciation processes [1-7]. Well-known examples are the often species-specific genitalia of spiders and insects [8-10], weapons involved in male-male competition over access to females [11,12], or ornamentation characters influencing female choice [13-15]. A trait complex that might evolve even more quickly than sexually dimorphic morphological characters is courtship behaviour, which might be modified under sexual selection long before morphological adaptations take place [16-20]. While the diversifying role of sexual selection in shaping courtship behaviour has been extensively studied, the role of evolutionary constraints has gained less attention [17,21-23]. The most obvious mode of constrained evolution is irreversibility, or the fact that some traits once lost cannot be reacquired. This is sometimes referred to as Dollo's law, and the characters involved as Dollo characters [21,24]. Typically, Dollo characters are either very complex or involve modifications at different levels, e.g. morphology and behaviour. Courtship related traits could represent such character systems, often involving specialized morphological adaptations and different behavioural components from both the male and female sex. Under the constraint argumentation, courtship characters, as long as they invoke also morphological adaptation, might thus evolve rather slowly, and moreover follow restricted pathways, with some characters only being lost and never reacquired during the evolutionary history of a group.

Courtship behaviour has been studied extensively in a wide range of taxa, but with a strong bias towards vertebrates. To understand the role of sexual selection in the diversification of life, it is important to study especially diverse groups, such as the insect order Hymenoptera. In this order, many species from a wide range of families have peculiar, male-specific modifications of the antennae [25-32]. Many such modifications have been shown to be connected to internal glands secreting a contact pheromone, and in some species, there is experimental evidence for antennal courtship mediating mate acceptance [26,33]. However, descriptions of hymenopteran courtship behaviour are rather scarce. In most species, courtship can only be observed if virgin females are at hand, which often requires rearing. In parasitoids, which account for the vast majority of hymenopteran species, this may also necessitate rearing the host as well. This is either very intricate or even impossible for species with unknown host relationships. Courtship data are consequently very sporadic and biased towards those species that can easily be cultured [29,34-44]. However, to conduct comparative studies and to reconstruct the evolution of courtship behaviour, an extensive taxon sampling is needed.

Recently, Steiner et al. [45] proposed a simple method to reproduce a specific form of courtship-related antennation, i.e. antennal coiling, in museum specimens. This method relies on the fact that the movement of the antennal flagellum is achieved by differences in haemolymph pressure and in the elasticity of the intersegmental membranes, since muscles are missing from flagelliform antennae of all insects. This coiling test acts as a proxy for courtship behaviour, as demonstrated in four hymenopteran species with known courtship behaviour [26,45,46]. Here we use this test to obtain antennal coiling data for 56 species of Diplazontinae, and examine the antennal morphology of a total of 64 species. Compared to other ichneumonid subfamilies [27], the Diplazontinae exhibit a large variation in the morphology and location of male-specific antennal structures, the so-called "tyloids". Using a well supported phylogeny of the group [[47], unpublished data], we assess the correlation between tyloids and antennal coiling in a comparative framework. We reconstruct the evolutionary history of tyloid morphology, tyloid location and antennal coiling under maximum parsimony, maximum likelihood and Bayesian approaches. Finally, we investigate whether the tyloid/antennal coiling character system shows signs of accelerated or of constrained evolution on the phylogeny.

## Results

### Variation in tyloid morphology and coiling behaviour

**Figure 1:**
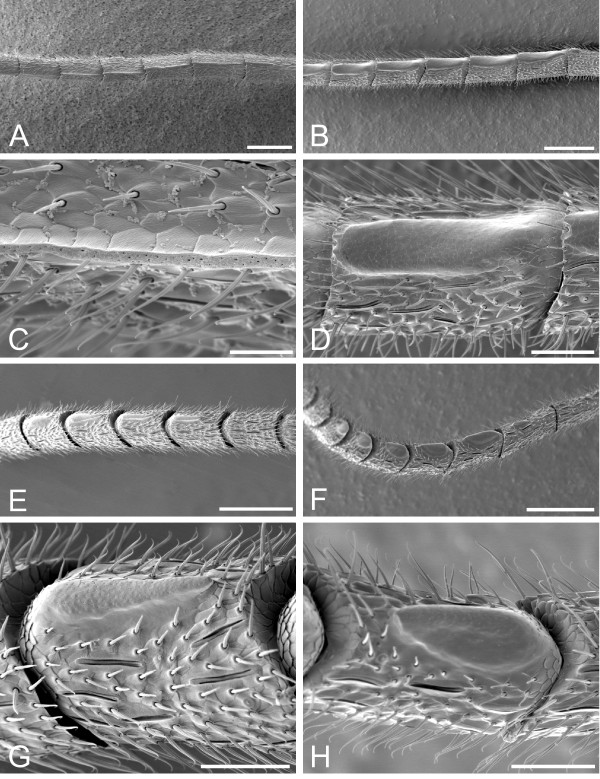
**Variation in tyloid morphology in Diplazontinae**. Shape of tyloids found in four species of diplazontine wasps. (A) and (C) *Syrphoctonus tarsatorius*, antennal segments 9 to 15 (A) and 10 (C), showing narrow, linear tyloids with abundant pores. Scale bars = 200 μm and 30 μm. (B) and (D) *Enizemum ornatum*, antennal segments 8 to 14 (B) and 10 (D), showing broad, linear tyloids with no pores. Scale bars = 200 μm and 50 μm. (E) and (G) *Sussaba erigator*, antennal segments 6 to 12 (E) and 8 (G), showing drop-shaped tyloids with pores. Scale bars = 200 μm and 50 μm. (F) and (G) *Sussaba pulchella*, antennal segments 6 to 14 (F) and 10 (G), showing oval, two-coloured tyloids with pores. Scale bars = 200 μm and 50 μm.

Of the 64 diplazontine species studied here, 34 have male-specific antennal modifications (Table 1). These tyloids show a large variability in terms of their shape, coloration and location on the antenna (Fig. 1, Table 2), with the shape ranging from narrowly linear tyloids, stretching over the whole length of the antennal segment, to broadly oval or drop-shaped, short tyloids (Table 1). Most of the tyloids were plain coloured, usually sharing the colour of the surrounding area, but some species possess two-coloured tyloids with a yellow and a dark brown half. In other ichneumonid and braconid subfamilies, only a single or at most two different types of tyloids can be found [27]. SEM investigations revealed numerous pores on the surface of those tyloids in 7 of 8 examined species with tyloids. In *Enizemum ornatum*, a species with broadly linear tyloids specific to the genus, we could not detect any pores (Figs. 1B and 1D); this brings into question whether the tyloids in this species are also used to transfer a secretion during courtship, or whether they rather serve for mechanical stimulation. The number and positions of tyloids on the antennae are correlated, with a low number of tyloids always being located on the middle segments of the antennae, and larger numbers of tyloids either ranging from the basal segments to the middle or from the middle close to the apex (Table 1).

**Table 1:** Taxon sampling, tyloid morphology and coiling behaviour

Species	SEM	N(m)	N(f)	# Tyloids	Tyloid type	Tyloid location	Coiling
Bioblapsis cultiformis		1		8	linear, narrow, whole length of antennomere, plain-coloured	apical	double-coiling
Campocraspedon annulitarsis		1		0	absent	-	no coiling
Campocraspedon caudatus	SEM	1		0	absent	-	no coiling
Diplazon annulatus		1		0	absent	-	no coiling
Diplazon deletus		1		0	absent	-	no coiling
Diplazon hyperboreus		2		0	absent	-	no coiling
Diplazon laetatorius		0		0	absent	-	?
Diplazon neoalpinus		0		0	absent	-	?
Diplazon orientalis		1		0	absent	-	no coiling
Diplazon pallicoxa		0		0*	absent*	-	?
Diplazon pectoratorius	SEM	2	1	0	absent	-	no coiling
Diplazon scutatorius		2		0	absent	-	no coiling
Diplazon tetragonus		1		0	absent	-	no coiling
Diplazon tibiatorius		1		0	absent	-	no coiling
Diplazon varicoxa		1		0	absent	-	no coiling
Diplazon zetteli		2		0	absent	-	no coiling
Diplazon sp. D		2		0	absent	-	no coiling
Enizemum ornatum	SEM	1		8	linear, broad, whole length of antennomere, plain-coloured	apical	double-coiling
Fossatyloides gracilentus	SEM	1		4	linear, narrow, whole length of antennomere, plain-coloured, with adjacent hole	middle	single-coiling
Homotropus crassicornis		0		11	linear, narrow, whole length of antennomere, plain-coloured	basal	?
Homotropus crassicrus		1	1	9	linear, narrow, whole length of antennomere, plain-coloured	apical	double-coiling
Homotropus elegans		1		9	linear, narrow, whole length of antennomere, plain-coloured	apical	double-coiling
Homotropus longiventris		1		8	linear, narrow, whole length of antennomere, plain-coloured	apical	double-coiling
Homotropus melanogaster		2	1	8	linear, narrow, whole length of antennomere, plain-coloured	apical	double-coiling
Homotropus nigritarsus		1		8	linear, narrow, whole length of antennomere, plain-coloured	apical	double-coiling
Homotropus nigrolineatus		2		8	linear, narrow, whole length of antennomere, plain-coloured	apical	double-coiling
Homotropus pallipes		1		8	linear, narrow, whole length of antennomere, plain-coloured	apical	double-coiling
Homotropus pictus	SEM	1		8	linear, narrow, whole length of antennomere, plain-coloured	apical	double-coiling
Homotropus signatus		2		8	linear, narrow, whole length of antennomere, plain-coloured	apical	double-coiling
Homotropus cf. lissosoma		1		6	linear, narrow, whole length of antennomere, plain-coloured	apical	double-coiling
Homotropus subopacus		0		8	linear, narrow, whole length of antennomere, plain-coloured	apical	?
Homotropus vitreus		1		4	linear, narrow, whole length of antennomere, plain-coloured	middle	single-coiling
Phthorima compressa		1		9	linear, narrow, whole length of antennomere, plain-coloured	apical	double-coiling
Phthorima xanthaspis		2		8	linear, narrow, whole length of antennomere, plain-coloured	apical	double-coiling
Promethes bridgmani		1	1	5	linear, narrow, whole length of antennomere, plain-coloured	middle	single-coiling
Promethes melanaspis		1		4	linear, narrow, whole length of antennomere, plain-coloured	middle	single-coiling
Promethes nigriventris		0		4	linear, narrow, whole length of antennomere, plain-coloured	middle	?
Promethes sulcator		1		3	linear, narrow, whole length of antennomere, plain-coloured	middle	single-coiling
Sussaba aciculata	SEM	1		4	drop-shaped, shorter than antennomere, plain-coloured	middle	single-coiling
Sussaba tertia		1		4	oval, broad, shorter than antennomer, two-coloured	middle	single-coiling
Sussaba cognata	SEM	10	2	0	absent	-	single-coiling
Sussaba dorsalis		1		5	oval, broad, shorter than antennomer, two-coloured	middle	single-coiling
Sussaba erigator	SEM	20	1	5	drop-shaped, shorter than antennomere, plain-coloured	middle	single-coiling
Sussaba flavipes	SEM	6	3	4	drop-shaped, shorter than antennomere, plain-coloured	middle	single-coiling
Sussaba placita		1		3	linear, narrow, shorter than antennomere, plain-coloured	middle	single-coiling
Sussaba pulchella	SEM	10	5	5	oval, broad, shorter than antennomer, two-coloured	middle	single-coiling
Sussaba punctiventris		0	5	6	linear, narrow, shorter than antennomere, plain-coloured	middle	?
Sussaba roberti		1		4	drop-shaped, shorter than antennomere, plain-coloured	middle	single-coiling
Syrphoctonus desvignesii		1		11	linear, narrow, whole length of antennomere, plain-coloured	apical	double-coiling
Syrphoctonus fissorius		0		10	linear, narrow, whole length of antennomere, plain-coloured	basal	?
Syrphoctonus tarsatorius	SEM	4	1	9	linear, narrow, whole length of antennomere, plain-coloured	apical	double-coiling
Syrphophilus asperatus		1		0	absent	-	no coiling
Syrphophilus bizonarius		1		0	absent	-	no coiling
Syrphophilus tricinctorius	SEM	1		0	absent	-	no coiling
Syrphophilus tricinctus		1		0	absent	-	no coiling
Tymmophorus erythrozonus		1		0	absent	-	no coiling
Tymmophorus obscuripes		1		0	absent	-	no coiling
Tymmophorus suspiciosus		1		0	absent	-	no coiling
Woldstedtius biguttatus		1		0	absent	-	no coiling
Woldstedtius citropectoralis		1	1	0	absent	-	no coiling
Woldstedtius flavolineatus		1		0	absent	-	no coiling
Woldstedtius holarcticus		1		0	absent	-	no coiling
Woldstedtius sp. A		2		0	absent	-	no coiling
Xestopelta gracilima		1		0	absent	-	no coiling

Abbreviations: N(m)/N(f): number of individuals of the two sexes subjected to the antennal coiling test. # Tyloids: number of antennal segments carrying tyloids. Tyloid type: tyloid type described from shape, length and colour. Tyloid location: location of the tyloid-bearing antennal segments on the antennae; basal = segments 4 to about10, middle = segments 8 to 13, apical = segments 9 to 17. Coiling: coiling type as described in the materials and methods section. *information obtained from Manukyan 1987.

**Table 2:** Ancestral state reconstruction of tyloid morphology and location on the antenna in the ancestors of Diplazontinae and of the three genus groups.

Trait		Diplazontinae	*Sussaba *group	*Syrphoctonus *group	*Diplazon *group
absence/presence	absent	0.00/0.01	0.00/0.00	0.00/0.00	0.99/1.0
	present	1.00/0.99	1.00/1.00	1.00/1.00	0.0/0.0

shape	absent	0.05/0.02	0.03/0.01	0.02/0.00	0.99/0.90
	narrow-linear	0.91/0.40	0.91/0.31	0.98/0.60	0.01/0.00
	broad-linear	0.01/0.17	0.00/0.14	0.00/0.21	0.0/0.04
	drop-shaped	0.01/0.16	0.01/0.18	0.00/0.12	0.0/0.04
	oval	0.03/0.26	0.06/0.36	0.00/0.07	0.0/0.02

length	absent	0.09/0.01	0.05/0.01	0.01/0.00	0.99/0.99
	short	0.09/0.08	0.17/0.32	0.00/0.00	0.00/0.00
	long	0.83/0.91	0.79/0.67	0.99/1.00	0.01/0.00

colour	plain	1.00/1.00	1.00/1.00	1.00/1.00	1.00/1.00
	two-coloured	0.00/0.00	0.00/0.00	0.00/0.00	0.00/0.00

location	absent	0.19/0.02	0.03/0.01	0.07/0.00	0.99/0.99
	middle	0.43/0.27	0.90/0.93	0.02/0.00	0.00/0.00
	basal	0.34/0.63	0.06/0.03	0.89/0.95	0.01/0.00
	apical	0.04/0.08	0.01/0.03	0.01/0.04	0.00/0.01

The probability of observing the indicated trait at the ancestral node of the specified groups is given first as resulting from the maximum likelihood and second from the Bayesian analysis.

**Figure 2:**
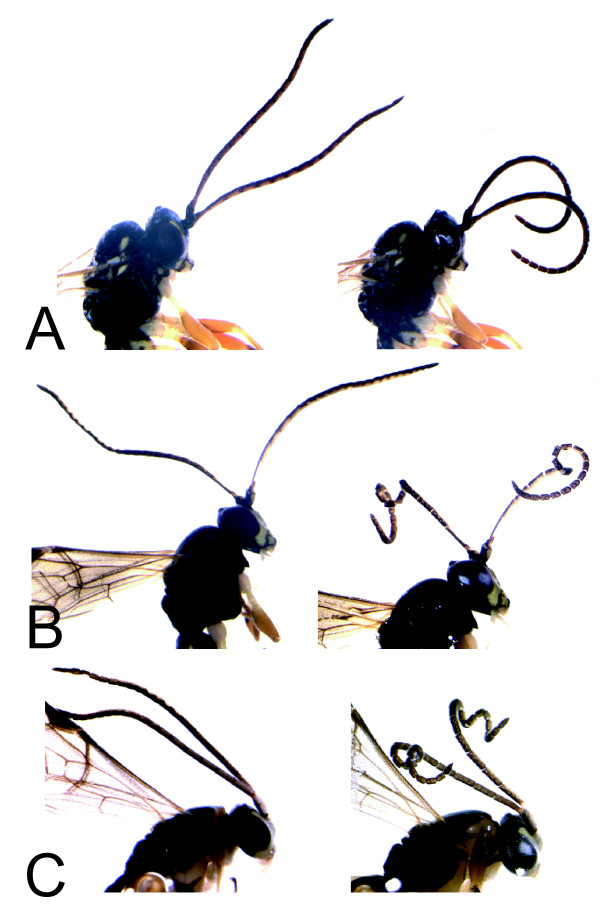
**Antennal coiling test applied to three diplazontine species**. The change in configuration of the antennae is shown in three species when transferred from absolute ethanol (left column) to distilled water (right column). (A) *Syrphophilus bizonarius*. (B) *Sussaba pulchella*. (C) *Syrphoctonus longiventris*.

We performed the coiling test with 22 female specimens from 11 species and 110 male specimens from 56 species. There was a clear sexual dimorphism in the reaction to the coiling test. While the antennae of all females bent slightly at the middle, the configurational changes observed in the male specimens varied a lot between genera and species. They ranged from an even curve of the antenna (no coiling, Fig. 2A), to a single, tight coil in the middle of the antenna (single-coiling, Fig. 2B) and two consecutive turns (double-coiling, Fig. 2C). Each coiling type was observed in at least two genera, and no intra-generic variation was observed except in the genus *Homotropus *(Table 1). The convex tyloids in all cases are located in a way that they face towards the inside of the coils, where they would be in close contact with the female antennae.

### Ancestral state reconstructions

Ancestral state reconstructions of tyloid morphology by maximum likelihood and Bayesian MCMC methods are similar, but with some differences especially in the reconstruction of the tyloid shape (Table 2). Both methods reveal that the most recent common ancestor of the studied diplazontines probably had narrowly linear, long and plain-coloured tyloids such as in *Promethes*, *Syrphoctonus*, *Homotropus *and related genera (Table 2). The location of those tyloids cannot be unambiguously reconstructed, with a middle position being favoured by maximum likelihood and an apical position favoured by the Bayesian analysis.

**Figure 3:**
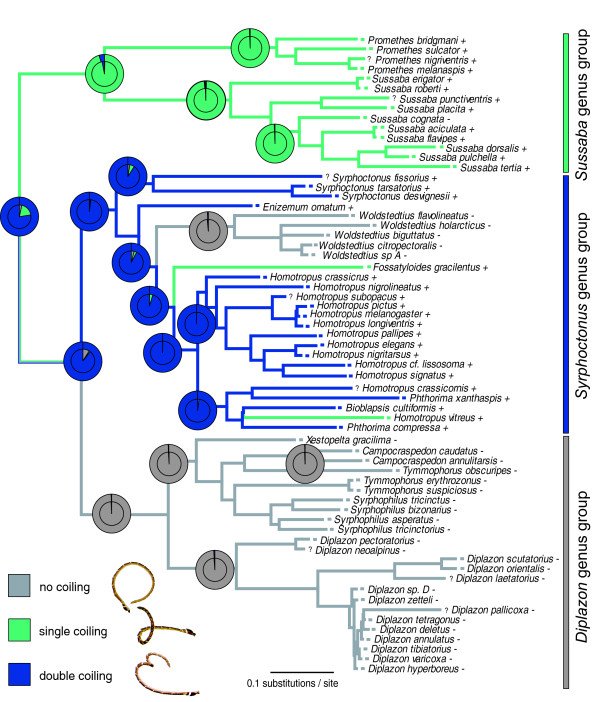
**Ancestral state reconstruction of antennal coiling in Diplazontinae**. Ancestral state reconstructions of antennal coiling is shown on top of the phylogeny. Parsimony reconstruction is shown as branch colour, with multi-coloured branches representing equally parsimonious solutions. Ancestral state reconstructions are shown as pie diagrams on each node of interest, with the outer circle representing maximum likelihood and the inner circles the Bayesian reconstruction. The character states found in the terminal taxa are indicated as squares of the respective colours, with question marks indicating species for which we could not obtain data on coiling behaviour. Symbols after taxon names indicate presence (+) or absence (-) of tyloids.

The MP, ML and Bayesian reconstructions of ancestral states in antennal coiling are shown in Figure 3. The three genus groups recognized by Klopfstein et al. [47] exhibit different types of antennal coiling. The members of the most basal *Sussaba *genus group all perform single-coiling. The ancestor of the *Syrphoctonus *clade probably performed double-coiling, with coiling subsequently lost in the genus *Woldstedtius *and transformed to single-coiling on two separate occasions, in *Fossatyloides gracilentus *and *Homotropus vitreus*. The *Diplazon *genus group probably lost any form of antennal coiling during courtship already in its stem lineage.

### The evolution of tyloids and antennal coiling

To test whether tyloids and antennal coiling evolved in a correlated manner, we compared the likelihoods of a model assuming independent evolution to a model assuming that the two traits co-evolve [48]. The likelihood ratio test revealed significant support for the dependent model (LHR = 15.43, df = 4, p = 0.0039). This result was confirmed by the Bayesian approach which converged on the dependent model with 99.98% posterior probability (Bayes factor = 9.8). The correlation between tyloid possession and antennal coiling was almost strict, with *Sussaba cognata *as the single exception. This species lacks tyloids or similar structures (as confirmed by SEM, Table 1), but consistently displays single coiling when examined with the coiling test.

**Figure 4:**
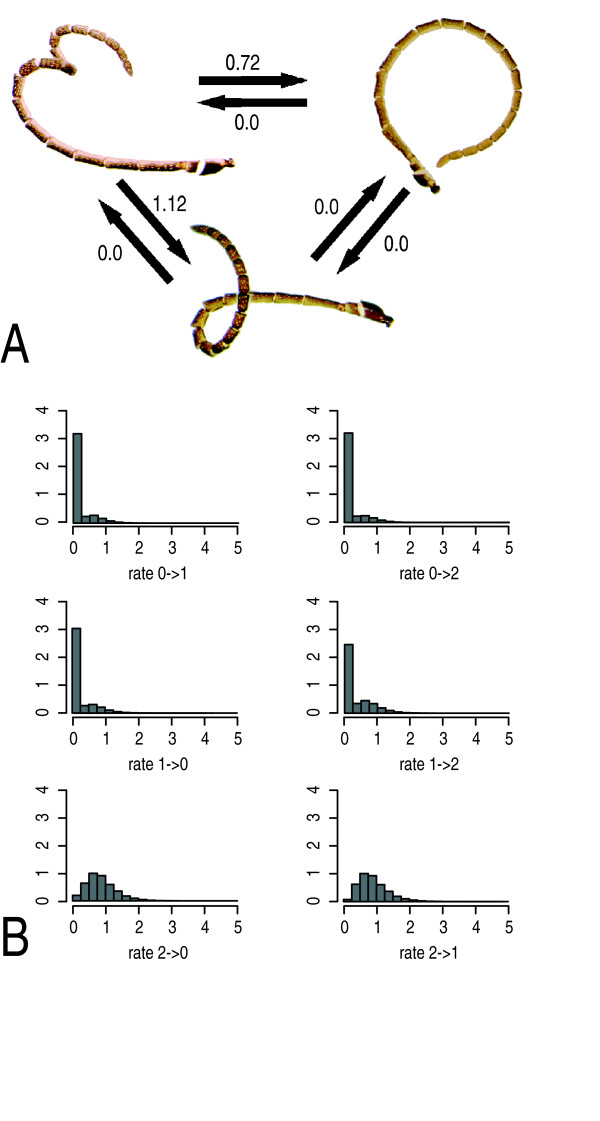
**Estimated transition rates between coiling states**. (A) Transition rates between the three states of coiling behaviour as estimated by maximum likelihood. (B) Posterior distribution of rates obtained from Bayesian analysis. States are: 0 = no coiling, 1 = single coiling, 2 = double coiling.

Overall, the rate of evolution of both morphological and behavioural characters was rather low, as indicated in Figure 3 for antennal coiling. The consistency (CI) and retention index (RI) of presence and absence of both tyloids and coiling behaviour indicated low levels of homoplasy (CI = 0.333 and RI = 0.931 for tyloids and CI = 0.500 and RI = 0.960 for coiling behaviour). To further study the mode of evolution of antennal coiling in Diplazontinae, we examined more closely the estimated transition rates between the two states, especially, whether the rate of change from the state 'absent' to 'present' (rate_0- > 1_) was equal to zero. To this end, we restricted the transition rates to being either all equal or one of them being zero, and compared the outcomes to the unrestricted case with two different transition rates. Under the unrestricted model, the rate_0- > 1 _was estimated to be zero, and this model had the highest likelihood. However, when compared to the equal rates model, this increase in likelihood was not significant with the likelihood ratio test (LRH = 1.61, df = 1, p = 0.20). The posterior probability of rate_0- > 1 _being zero was estimated as 72% by the MCMC approach, which integrates over phylogenetic uncertainty. When taking the mode of antennal coiling into account, ML estimations confirm a zero rate for the transitions 0- > 1 and 0- > 2, as shown in Figure 4A. The corresponding posterior probability distributions obtained from the Bayesian analysis are shown in Figure 4B.

## Discussion

We successfully applied the coiling test developed by Steiner et al. [45] to obtain courtship behavioural data for a large number of parasitoid wasp species to reconstruct the evolution of this trait. The underlying assumption was that the coiling test performed on dead specimens provides information about the mode of antennal coiling actually performed by the wasps. The observed sexual dimorphism in the outcome of the antennal coiling test supports this assumption, as does the high correspondence between the configurational change of the antenna and the movement actually performed by the males in *Syrphoctonus tarsatorius*. Three other hymenopterans for which information on antennal courtship is available further confirm this link [[45], unpublished results]. However, we have to bear in mind that species not showing any coiling in the test might still perform another form of antennal courtship, such as antennal tapping or stroking. Moreover, the link between the outcome of the test and the behaviour actually displayed during courtship might not be strict, as the coiling test only reflects the presence of the morphological requirements to perform antennal coiling. In *Sussaba cognata*, the only species in the *Sussaba *genus group that does not possess any tyloids but still performs antennal coiling in the coiling test, one could imagine that the morphological requirements for coiling have not yet been completely reduced, although the tyloids as release and spread structures for a courtship pheromone are absent. It would be of great interest to know whether the males of this species actually do or do not perform antennal coiling during courtship. Such data would provide insights into how the different aspects of the tyloid/antennal coiling system co-evolve, i.e. the behaviour of the males *per se*, the morphological prerequisites for the movement, the tyloids as release and spread structures for a sex pheromone and finally the preference of the females. The observation that not all aspects of this character complex need to be present in all species opens up the possibility of evolution through intermediate stages. *Enizemum ornatum *provides another such example. Although this species possesses tyloids, we could see no pores on their surface unlike in all the other species examined. The absence of tyloid pores might indicate that they no longer serve as release and spread structures for a courtship pheromone. Instead, they could be involved in purely mechanical stimulation in this species.

The strong correlation observed in diplazontines between tyloids and antennal coiling further supports the hypothesis that male-specific antennal modifications are likely involved in courtship [25,26,29,31-33,39,42,49]. Various forms of antennal courtship have been described in this diverse insect order, and even more diverse antennal modifications. As exemplified in the present study, the coiling test could be used to further establish the link between morphology and behaviour in the case of antennal courtship [45], and shed light on the evolution of courtship behaviour in this order.

The overall low rate of evolution and low levels of homoplasy in the courtship traits analyzed here seem to contradict previous assertions that characters under sexual selection would evolve at comparatively high rates [1-4,6,9]. However, there are numerous previous examples demonstrating that parts of courtship behaviour can evolve in a very conservative mode, translating in high phylogenetic informativeness of some courtship characters [50-53]. Although sexual selection can in some cases lead to accelerated evolution, this does not need to be the case, and might largely depend on the variability of a trait and on geographic and population dynamic patterns [16]. Moreover, it has been shown that female preference sometimes even imposes selection against a change in mating behaviour, thus decelerating the speed of evolution [e.g. [54]]. Courtship behavioural traits are accordingly influenced by a variety of selective pressures and evolutionary constraint which might differ between traits in a single species. In our case, the estimation of a zero rate of regain of antennal coiling suggests that this trait is under severe constraints and that its loss evolves as an irreversible character, at least over the timescale examined. This is further supported by the fact that the loss of antennal coiling involved 31 species in two groups, implying that there would have been plenty of time for a re-acquisition of the coiling state. On the other hand, there is a large variation found in antennal modifications in this subfamily, especially in the *Sussaba *genus group, implying that a more detailed examination of courtship behaviour in Diplazontinae might reveal a larger variation and higher evolutionary lability in other courtship traits.

The concept of irreversibility of evolution was already introduced by Dollo in 1893 [24], and has been extensively disputed in the past [e.g. [55,56]]. It has been invoked to explain patterns of evolution of complex morphological characters, of sex determination systems, polyploidy and parthenogenesis, of gains and losses of genes, and many others [56-59]. In our case, there are arguments both in favour and against antennal coiling being a Dollo character in the narrow sense. From a theoretical point of view, a complex trait involving different character systems is difficult to be regained, once it is lost, and this description clearly applies to antennal coiling. On the other hand, there might be some pre-adaptations that could favour the reacquisition of some form of antennal courtship in parasitoid hymenopterans. For instance, the high sensitivity of the female antenna which is needed for host location and evaluation could facilitate the evolution of male behaviours that target the antenna of the female for sexual stimulation. Moreover, in the mounted position, the male body parts that can most easily access the female's antennae are the male's antennae. The variability of antennal courtship and related morphological adaptations in the order Hymenoptera further suggest that these behaviours might not all be homologous, but instead might represent convergent evolutionary events. Further studies on a larger evolutionary scale are needed to ascertain the irreversibility of a loss of antennal courtship in Hymenoptera.

## Conclusions

Antennal coiling is a mode of courtship found in many hymenopteran and even other insect species, and represents a complex character involving both morphological and behavioural adaptations. We here demonstrate in a comparative context that antennal courtship is highly correlated with the possession of sexually dimorphic antennal modification in diplazontine parasitoid wasps. Moreover, we show that antennal coiling evolves at a low rate. It is lost two times independently in the subfamily and never re-acquired, which is in accordance with Dollo's law of irreversibility of evolution.

## Methods

### Tyloid morphology

Presence and absence of tyloids and morphological types were recorded for 64 species of Diplazontinae, classifying all male-specific, convex antennal structures of a minimum size of one fifth of the diameter of an antennal segment as tyloids, following the conception of Diller [60]. To study the evolution of the tyloids, we classified them into different types, examining their number, length, shape and colour. To indicate the location of the tyloids on the antennae, we progressively numbered the antennal segments from the segment attached to the head capsule (scape) to the apex following Bin and Vinson [31]. The use of a stereo-microscope (Leica Wild M10) at magnification 80 proved sufficient to score these data; however, to make sure not to have overlooked tyloid-like structures and to study the ultrastructure, we further examined some species by scanning electron microscopy (SEM). To this end, the antennae of males stored in 80% ethanol were mounted, air dried and gold-sputtered. SEM studies were performed with a Philips XL30 FEG scanning electron microscope. No overlooked, tyloid-like structures were found by SEM analysis.

### Reproducing antennal coiling

Courtship behaviour has to date only been described in two diplazontine species [45,46]. To simulate a change of pressure inside the antennae and thus reproduce antennal courtship in museum specimens, we used a method developed by Steiner et al. [45]. We first cut off the antennae of dried specimens or of specimens kept in 80% ethanol. The amputated antennae were then put into 10% aqueous potassium hydroxide (KOH) for 10 min at room temperature to macerate the inter-segmentary membranes. Antennae from dried specimens were then put into 80% ethanol overnight. Afterwards, we placed them in 100% ethanol for 10 min and finally transferred them to distilled water. Because of different viscosities of the two liquids, this transfer resulted in an overpressure inside the antenna that led to an elongation of the inter-segmentary membranes and eventually to a configurational change. We recorded this change in the configuration of the antennal segments of male specimens of 56 diplazontine species (Table 1). To test reproducibility, we included multiple specimens for some of the species for which enough fresh material was available. Tests were regarded as successful if the antennae performed a distinct movement after being transferred to water, following the recommendations of Steiner et al. [45]. We classified those movements as antennal coiling that involved a specific range of segments coiled up in a spiral-like form. We denoted spirals of 200° to 360° as single-coils and spirals of more than 360° as double-coils.

### Reconstructing the evolution or antennal courtship

The test phylogeny employed in this study was obtained from Klopfstein et al. (unpublished data). It is based on four molecular markers, two mitochondrial (cytochrome oxidase subunit 1 and NADH 1) and two nuclear (28S, elongation factor 1-α). The phylogeny is well resolved, with most nodes highly supported. We used the tree with the highest likelihood as obtained from the partitioned Bayesian analysis for the maximum parsimony (MP) and maximum likelihood (ML) estimations, and 10'000 trees from the post-burnin Bayesian tree distribution for the MCMC methods. MP and ML reconstruction of ancestral states were conducted in Mesquite [61], ML under the Mk1 model of evolution. Additionally, rate parameters and ancestral states at each node of interest were reconstructed under a Bayesian approach using the program BayesMultiStates from the BayesTraits package [62]. In the Markov-chain Monte Carlo approaches, we applied an exponential reversible-jump hyperprior within the interval of zero to 30 and set the ratedev parameter, which controls the proposal of new rate values, to 8. This resulted in an acceptance rate between 20% and 29% in all analyses, which falls inside the recommended range. To draw inferences about the modes of evolution, we restricted the rate parameters to obtain the likelihoods of different models [63]. To test whether antennal coiling evolved as an irreversible character, we forced the rate parameters of the reversals to zero, and restricted the most ancestral node to the 'present' state, following Goldberg and Igic [64]. The resulting likelihood values were then compared under the likelihood ratio test [63]. Alternatively, the posterior distribution of the models was assessed using reversible-jump MCMC [48]. To test for correlated evolution of tyloids and coiling behaviour in diplazontine wasps, we used BayesDiscrete from the BayesTraits package [48] under an independent and a dependent model of evolution. Likelihoods obtained under the two models with 50 ML attempts per tree were compared by a likelihood ratio test. Posterior probabilities of the dependent and independent models, and harmonic means of the likelihoods for Bayes factor comparisons, were obtained by reverse-jump MCMC, using the above-mentioned parameter settings.

## Authors' contributions

SK identified the Diplazontinae species, documented the tyloid morphology, performed the coiling tests and did the comparative analyses. DLJQ contributed to the discussion of results and to the interpretation of the phylogenetic inferences of character evolution. CK contributed to the functional interpretation of the sexually dimorphic characters and to the conception of this research. All authors revised the manuscript drafts, read and approved the final manuscript.
